# Supplementary material for: Financial health as a measurable social determinant of health
Source: PLoS One. 2020 May 18;15(5):e0233359. doi: 10.1371/journal.pone.0233359 (PMC7233592; doi:10.1371/journal.pone.0233359)
Supplement: Attachment — Submitted filename: Responses to Reviewers.docx [file pone.0233359.s003.docx]

| Reviewer #1: The manuscript has been considerably improved and sophisticated. I have a few questions as follows:  1. Research question and Discussion  The answer to the first research question (i.e., introduce a construct of financial health into the domain of public health defined by more than just individual measures of economic hardship) is not clearly stated in the discussion section. Please add more explanations why and which part of your analyses can be the evidence of the distinction between financial health and economic hardship (indicated by food security, housing security, and energy security). | Thank you for the opportunity to provide more clarification. We added text in the discussion (please see lines 295-297). |
| --- | --- |
| 2. “To avoid collinearity due to the interrelated nature of energy security, housing security and food security, only questions relating to energy security were used for the PCA (p.10, line 119-201)”. If only energy security items were included in the PCA, how do we know financial health variables are distinct from housing security and food security? | Thank you for this insight. The Spearman correlation tests between each domain of our financial health construct (as well as the overall score) and housing and food insecurity were provided as demonstration construct validity to show that financial health is distinct from housing and food insecurity. The text that we added in response to your previous question will likely help us to clarify for readers the intention of these results. |
| 3. As the sample represents low-income caregivers of young children, it would be more valuable if the discussion provides implications beneficial to this specific population. | Thank you for this point. Please see lines 321-330. We have added narrative at the end of the discussion section (lines 332-339) that describes the implications of this study to benefit low-income families with young children. |
| Reviewer #2: Thank you for addressing my issues. The paper does need a bit of copy editing. This may be something the editorial office can help with (e.g., some of the discussion around the new logistic regression model was oddly written). Other than that, the additions to the paper were appropriate. | Thank you for the opportunity to correct these, we have taken time for more careful copyediting. |
